# Numerical study of virus transmission through droplets from sneezing in a cafeteria

**DOI:** 10.1063/5.0040803

**Published:** 2021-02-25

**Authors:** Liangyu Wu, Xiangdong Liu, Feng Yao, Yongping Chen

**Affiliations:** 1 College of Electrical, Energy and Power Engineering, Yangzhou University, Yangzhou 225009, People's Republic of China; 2 Jiangsu Key Laboratory of Micro and Nano Heat Fluid Flow Technology and Energy Application, School of Environmental Science and Engineering, Suzhou University of Science and Technology, Suzhou 215009, People's Republic of China; 3 Key Laboratory of Energy Thermal Conversion and Control of Ministry of Education, School of Energy and Environment, Southeast University, Nanjing, Jiangsu 210096, People's Republic of China

## Abstract

To provide a comprehensive understanding of virus transmission inside small indoor spaces, numerical simulation of sneezing droplets spreading in a cafeteria is conducted through computational fluid dynamics. The numerical results show that dining face to face is extremely vulnerable to direct infection by others' respiratory droplets. Different heights of droplet sources are compared, which indicates that sneezing from a standing person results in a longer survival time of droplets in the air. Scenarios with fewer customers without face to face seating and turning off the horizontal supplying air conditioner are examined as well. Various surfaces are still detected with droplets in 300 s after sneezing. The horizontal supplying air conditioner causes increment in the velocities of the droplets and leads to further spreading of the droplets. It is essential to sanitize all surfaces in a cafeteria including the walls, floor, ceiling, and tables that are not occupied by any customer. Keeping a safe distance in small indoor spaces such as cafeterias does not offer sufficient protection for activities without wearing a face mask. It is recommended that cafeterias and canteens only accept take-away orders.

## I. INTRODUCTION

During the past 12 months, the COVID-19 pandemic caused by the novel coronavirus “SARS-CoV-2” has led to over 70 × 10^6^ infections and more than 1.6 × 10^6^ mortalities worldwide.^1^ It is acknowledged that respiratory droplets are the main route that SARS-CoV-2 infects humans.^2^ Thousands of droplets can be produced during sneezing or coughing,^3^ and maximum ∼10^8^ copies of virions can be laden in 1 ml of droplets.^4^ Besides, there are a lot of human activities that generate droplets and aerosols^5–12^ such as dentistry that special precautions should be taken^13^ to suppress the spreading of viruses. Moreover, the deposition of droplets on surfaces around the sneezing person increases the risk of infecting others through contacting these surfaces.^2^ Also, it is essential to determine the drying time of virus laden droplets fallen on surfaces around the infected person to prevent infecting through contacting these contaminated surfaces. The drying time is found to be strongly affected by humidity, ambient temperature, and surface wettability.^14^ According to the further computational study of Bhardwaj and Agrawal, the drying time of a spread droplet on a surface is largely extended when the liquid film thins to nanometer. This explains why the coronavirus survives on a surface for days.^15^ In addition, respiratory droplets can dry in a short time on hydrophilic surfaces, which are preferable for the design of personal protection equipment surfaces.^16^ Since it is impossible to stop indoor activities, it is of significance to reduce the droplets in the indoor environment where the droplets can survive for a longer time than in the outdoor environment.^17^

In this context, an in-depth understanding of the transmission characteristics of droplets and aerosols in an indoor environment is essential. It is found that during each minute of speaking, approximately 20 000 particles in the range of 0.8 *μ*m–5.5 *μ*m are released, which can load over 100 000 copies of virions.^18,19^ According to this, the numerical simulation of Abuhegazy *et al.*^20^ shows that over 180 droplets can be transmitted to a neighbor student in a classroom even though the distance between students is 2.4 m. It is recommended to keep the window open and use glass screens in front of students in classrooms. Transmission of coughing droplets from a walking person is simulated by Li *et al.*, and the effect of space size is examined in particular. Two distinct modes are discovered that the cloud of droplets after coughing detaches or attaches depending on the walking velocity and space size.^21^ The motion of respiratory droplets depends on their sizes, and the critical diameter is reported by Wang *et al.* Large droplets (diameter ≥ 100 *μ*m) fall on the ground before they evaporate to droplet nuclei. While droplets smaller than 50 *μ*m evaporate to droplet nuclei in front of the coughing person, droplets between 50 and 100 *μ*m evaporate to droplet nuclei in the ambient air during trasmission.^22^ A calculation model is proposed by Chaudhuri *et al.*, which identifies the probability of infection caused by inhaling droplets loaded with virus. Droplets with a diameter between 10 and 50 *μ*m are found to be most infectious.^23^ The condition of air flow around the respiratory droplets is essential in the transmission process. The life of droplets is found to be extended due to the vortex of the expiratory cloud and hence can remain airborne for a longer time than expected.^24^ According to Wang *et al.*,^25^ the bottom-in and top-out air distribution is a favorable ventilation strategy in hospital wards to prevent cross-infection. This is in contrast to what was believed by the public that the bigger droplets can even spread to a longer distance than the smaller ones in a room.^26^ Moreover, the safe distance must be increased to 4 m when someone sneezes.

Wearing a mask is significantly helpful for suppressing the transmission of droplets.^27–29^ The traveling distance of the droplets can be reduced by half if there is a mask in front of a coughing mouth.^29^ However, there are certain situations that people do not wear a mask, for example, during dining.^30^ In a lot of regions, indoor dining is still going on. Although various measures have been proposed^31–34^ for maintaining a clean indoor environment, most measures do not consider the transmission characteristics of the airborne droplets and aerosols. The detailed information on the movement, trajectories, and deposition of the droplets is useful in making guidelines that prevent people from infection during indoor activities.

Therefore, the virus transmission of respiratory droplets from sneezing in a small cafeteria with 6 tables is studied numerically. The distribution of droplets after sneezing is examined in 6 scenarios. The deposition of droplets on different indoor surfaces is discussed. The mass variation of droplets floating in air is analyzed. The strategy of lessening the number of customers and advising separate seats for customers is examined. In addition, the influence of the air flow from a horizontal supplying air conditioner is studied to provide a guideline for the ventilation in small cafeteria and other small indoor spaces.

## II. MATHEMATICAL MODEL AND NUMERICAL SOLUTION

**FIG. 1.**
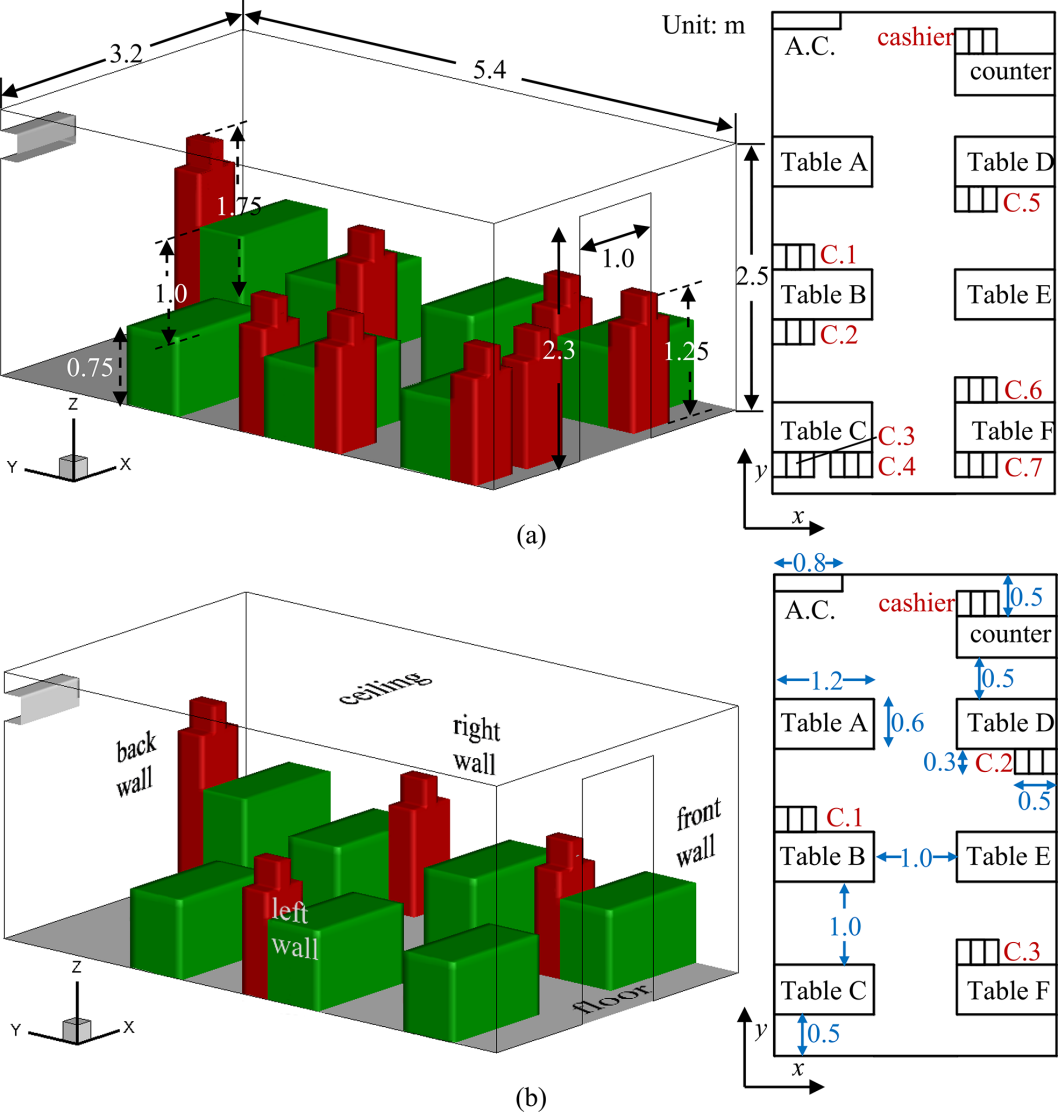
Schematic of a cafeteria with customers: (a) scenario with face to face dining and (b) scenario without face to face dining.

Transmission of droplets from sneezing in a small cafeteria with 6 tables (marked as Tables A to F in Fig. 1) and a counter is studied in a three-dimensional indoor space with length × width × height = 5.8 m × 3.2 m × 2.5 m. A cashier is standing behind the counter, and the maximum capacity of the cafeteria is 24 customers. There is an opening door on the front wall of the cafeteria (width × height = 1 m × 2.3 m) and no opening window on other walls, which is common in a lot of small cafeterias and fast-food restaurants. An air-conditioner (AC) mounted on the top of the back wall heats the indoor air, and the heated air is supplied along the horizontal direction (*y*-negative). The size of the table is 1.2 m × 0.6 m, and the interval between tables is 1 m in both *x* and *y* directions. The human bodies are simplified as a combination of a cuboid body with a cuboid head. The cashier standing behind the counter is 1.75 m tall. The height of the seated customers is 1.25 m. For both the cashier and customers, the cross section of their heads in the *xy* planes is 0.17 m × 0.3 m, and the cross section of their bodies is 0.5 m × 0.3 m. A rectangle moth-print (4 cm × 1 cm) is utilized according to the study of Dbouk and Drikakis.^35^

Normally, most of the seats in the cafeteria are occupied by customers during the busy hours. Some customers may sit face to face across one table during dining [Fig. 1(a)]. After the outbreak of COVID-19, some restaurants and cafeterias recommend the customers to seat separately that one table is occupied by one customer exclusively. In some cafeterias and canteens, especially those in the universities in China, customers are recommended to take seats that all facing the same direction during dining. Hence, the effect of advising customers dining without sitting face to face is examined using the model shown in Fig. 1(b).

### A. Governing equations for turbulent flow

The velocity of the air out from a sneezing mouth is set as 50 m/s in this work.^25^ The Reynolds number is 1.35 × 10^4^, indicating turbulent flow conditions, which agrees with other studies.^36–38^ Therefore, the realizable *k*-*ε* model^39,40^ is used to obtain accurate results for the air flow in the cafeteria within acceptable computational consumptions. The turbulence kinetic energy *k* and its rate of dissipation *ε* are solved using

$$\frac{\partial }{\partial t}(\rho k)+\frac{\partial }{\partial x_{j}}(\rho ku_{j})=\frac{\partial }{\partial x_{j}}[(\mu +\frac{\mu_{t}}{\sigma_{k}})\frac{\partial k}{\partial x_{j}}]+G_{k}+G_{b}-\rho \varepsilon -Y_{M}+S_{k},$$ (1)

$$\begin{array}{c} \frac{\partial }{\partial t}(\rho \varepsilon )+\frac{\partial }{\partial x_{j}}(\rho \varepsilon u_{j})=\frac{\partial }{\partial x_{j}}[(\mu +\frac{\mu_{t}}{\sigma_{\varepsilon }})\frac{\partial \varepsilon }{\partial x_{j}}]+\rho C_{1}S\varepsilon -\rho C_{2}\frac{\varepsilon^{2}}{k+\sqrt{\nu \varepsilon }}+C_{1\varepsilon }\frac{\varepsilon }{k}C_{3\varepsilon }G_{b}+S_{\varepsilon }, \end{array}$$ (2)

in which the eddy viscosity is calculated using

$$\mu_{t}=\rho C_{\mu }\frac{k^{2}}{\varepsilon }.$$ (3)

In the realizable *k*-*ε* model, the parameter *C_μ_* is a function of the mean strain, rotation rates, angular velocity of the system rotation, and turbulence fields,

$$C_{\mu }=\frac{1}{A_{0}+A_{S}\frac{kU^{*}}{\varepsilon }},$$ (4)

where $U^{*}=\sqrt{S_{ij}S_{ij}+{\tilde{\Omega }}_{ij}{\tilde{\Omega }}_{ij}}$, ${\tilde{\Omega }}_{ij}=\Omega_{ij}-2\varepsilon_{\mathrm{ijk}}\omega_{k},\Omega_{ij}=\bar{\Omega_{ij}}-\varepsilon_{\mathrm{ijk}}\omega_{k}$, and *A*_0_ and *A*_S_ are constants.

The values and physical meanings of the coefficients in Eqs. (1)–(4) are listed in Table I.

**TABLE I.** Coefficients in the governing equations.

Coefficients	Physical meanings
$C_{1}=\text{max}[0.43,\frac{\eta }{\eta +5}]$, $\eta =S\frac{k}{\varepsilon }$, $S=\sqrt{2S_{ij}S_{ij}}$ C_2_ = 1.9, C_1__*ε*_ = 1.44	Constants
$G_{k}=-\bar{\rho u_{i}^{\prime }u_{j}^{\prime }}\frac{\partial u_{j}}{\partial x_{i}}$	The generation of turbulence kinetic energy due to the mean velocity gradients
$G_{b}=\rho g\frac{\mu_{t}}{Pr_{t}}\frac{\partial T}{\partial x_{i}}$	The generation of turbulence kinetic energy due to buoyancy force
$Y_{M}=2\rho \varepsilon M_{t}^{2},M_{t}=\sqrt{\frac{k}{a^{2}}},a\equiv \sqrt{\gamma RT}$	The contribution of the fluctuating dilatation in compressible turbulence to the overall dissipation rate
*σ*_k_ = 1.0, *σ*_ε_ = 12	Turbulent Prandtl numbers for *k* and *ε*
C_3__*ε*_	The buoyancy effect on *ε*
*S*_k_, *S*_ε_	Source terms
$\bar{\Omega_{ij}}$	Mean rate-of-rotation tensor viewed in a moving reference frame with the angular velocity *ω*_k_

### B. Discrete phase model for droplets

The trajectories of the respiratory droplets are calculated by the discrete phase model (DPM), which has been proven capable of predicting the movement of droplets from human sneezing by Dbouk and Drikakis.^35^

In the DPM model, the trajectory of a droplet, which is regarded as a particle, can be obtained by integrating the force balance in a Lagrangian reference frame,

$$m_{p}\frac{d\vec{u_{p}}}{dt}=m_{p}\frac{\vec{u}-\vec{u_{p}}}{\tau_{r}}+m_{p}\frac{g(\rho_{p}-\rho )}{\rho_{p}}+\vec{F},$$ (5)

where *m*_p_ is the particle mass, $\vec{u}$ is the velocity of air, $\vec{u}$ is the velocity of the particle, *ρ* is the density of air, *ρ*_p_ is the density of the droplet, $\vec{F}$ is the additional force caused by Brownian force and Saffman lift force, $\frac{\vec{u}-\vec{u_{p}}}{\tau_{r}}$ is the drag force, and *τ*_r_ is the relaxation time of the droplet calculated as follows:

τr=ρpdp218μ24CdRe, (6)

where *μ* is the molecular viscosity of air, *d*_p_ is the diameter of the droplet, and Re=ρpdp|up→−u→|μ is the relative Reynolds number.

### C. Boundary conditions

The cases are studied in winter conditions that the outdoor temperature is −4.1 °C and the setting temperature of the A.C. is 20 °C. The person sneezing is assumed to be infected with SARS-Cov-2 and having a fever with a body temperature of 39 °C. While the body temperature of other people inside the cafeteria is 36.5 °C, the heat flux from a human body to the environment is 1100 W/m^3^. The mass flow rate of the A.C. is 0.255 kg/h, and the gauge pressure at the door is 0 Pa. Natural convection of air caused by the temperature difference is considered. The sneeze lasts for 0.12 s, and the size distribution of the respiratory droplets is calculated according to the Rosin–Rammler distribution law.^41,42^

## III. SOLUTION METHODS

### A. Assumptions

Several assumptions are made in this work as follows:

(1) Heat and mass transfer between the droplets and the indoor air is neglected.
(2) Collision and breakup of the droplets are neglected.
(3) Air flow caused by human breathing is neglected.
(4) The movement of the human inside the cafeteria is not considered.

### B. Mesh independency analysis

Structured meshes with hexahedral cells are used in this work. To obtain accurate results, local meshes in front of the sneezing person are refined. The velocity magnitude and gauge pressure at location *x* = 1.6 m, *y* = 2.7 m, *z* = 1.25 m calculated using five different meshes are compared (Table II). It can be concluded that there is no obvious difference between the results obtained from M4 with 1 700 143 cells and M5 with 2 630 478 cells. Hence, the models using M4 are capable of predicting the fluid flow inside the cafeteria.

**TABLE II.** Mesh independence analysis at the center cafeteria.

Mesh	M1	M2	M3	M4	M5
Number of cells	451 196	681 188	1 414 508	1 700 143	2 630 478
Velocity magnitude (m/s)	0.058 8	0.081 7	0.064 8	0.063 1	0.063 0
Gauge pressure (Pa)	−0.005 97	−0.006 57	−0.007 27	−0.007 44	−0.007 42

### C. Numerical solution

The commercial CFD software Ansys Fluent is used as the solver for the governing equations based on the finite volume method. The third order MUSCL (Monotone Upstream-Centered Schemes for Conservation Laws) scheme^43^ is utilized in the discretization of the momentum equations, while the second order upwind scheme is used for other equations. The coupling between pressure and velocity is achieved using the PISO (pressure implicit with splitting of operators) algorithm.^44^ To reduce the computational consumption, the varying time step is applied.^45^ The time steps are Δ*t* = 0.001 s in 0 s–0.12 s, Δ*t* = 0.01 s in 0.12 s–1 s, Δ*t* = 0.1 s in 1 s–10 s, and Δ*t* = 1 s in 10 s –300 s.

## IV. RESULTS AND DISCUSSION

### A. Indoor airflow

**FIG. 2.**
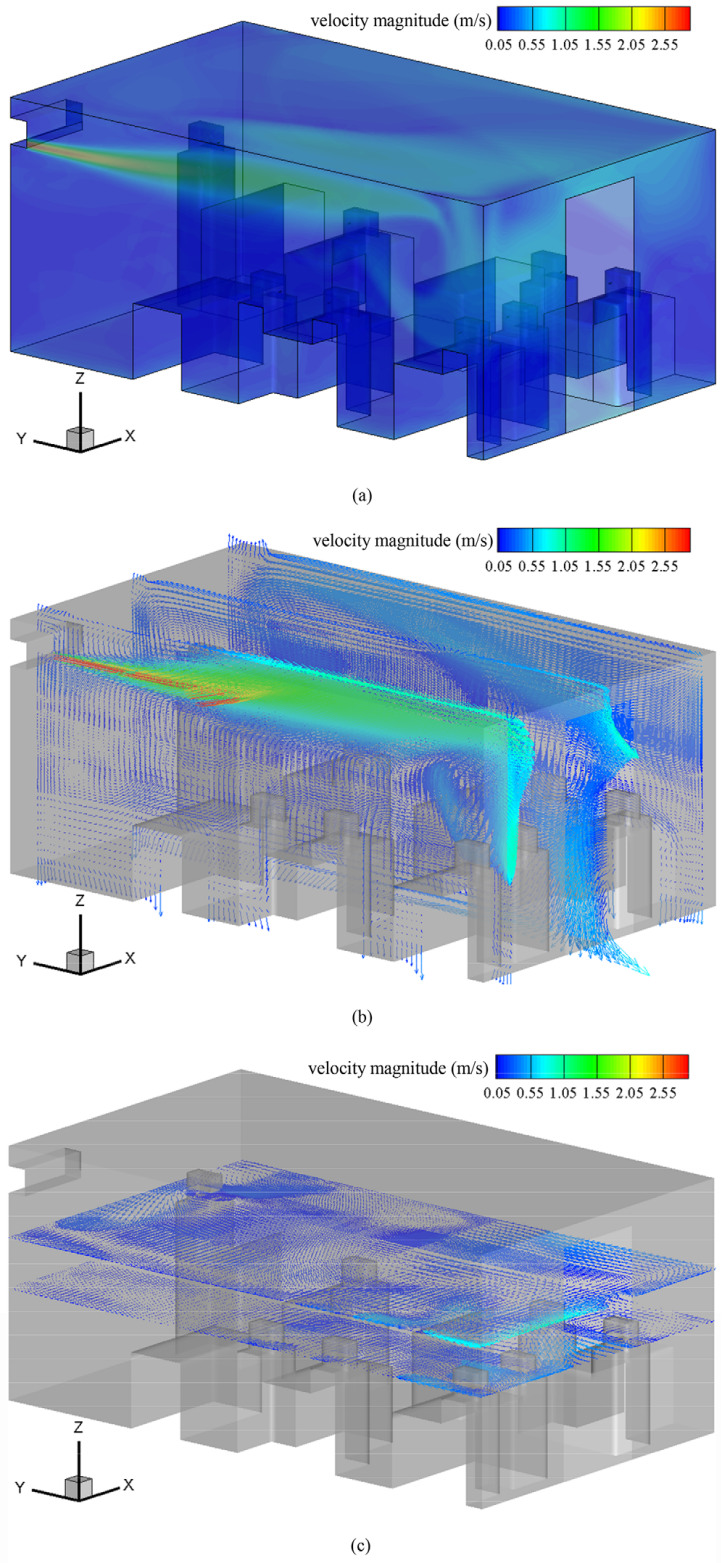
Indoor air flow (a) velocity magnitude, (b) velocity vectors in slices at *x* = 0.4 m, 1.8 m, and 3.2 m, and (c) velocity vectors in slices at *z* = 1.1 m and 1.6 m (air flow from the A.C. is along the y negative direction).

The fluid flow inside the cafeteria is the combined results of natural convection caused by the temperature gradient and forced convection sourcing from the A.C. The temperature of the human body is higher than the surrounding air so that the small scale of vortexes is observed around human bodies [Fig. 2(b)]. The A.C. attributes to strong circulating flow in *yz* planes [Fig. 2(c)] and causes a large scale of vortexes in the upper space inside the cafeteria. Both result in longer renewal of the local air, which is disadvantageous in obtaining a clean indoor environment.

### B. Transmission of droplets

**FIG. 3.**
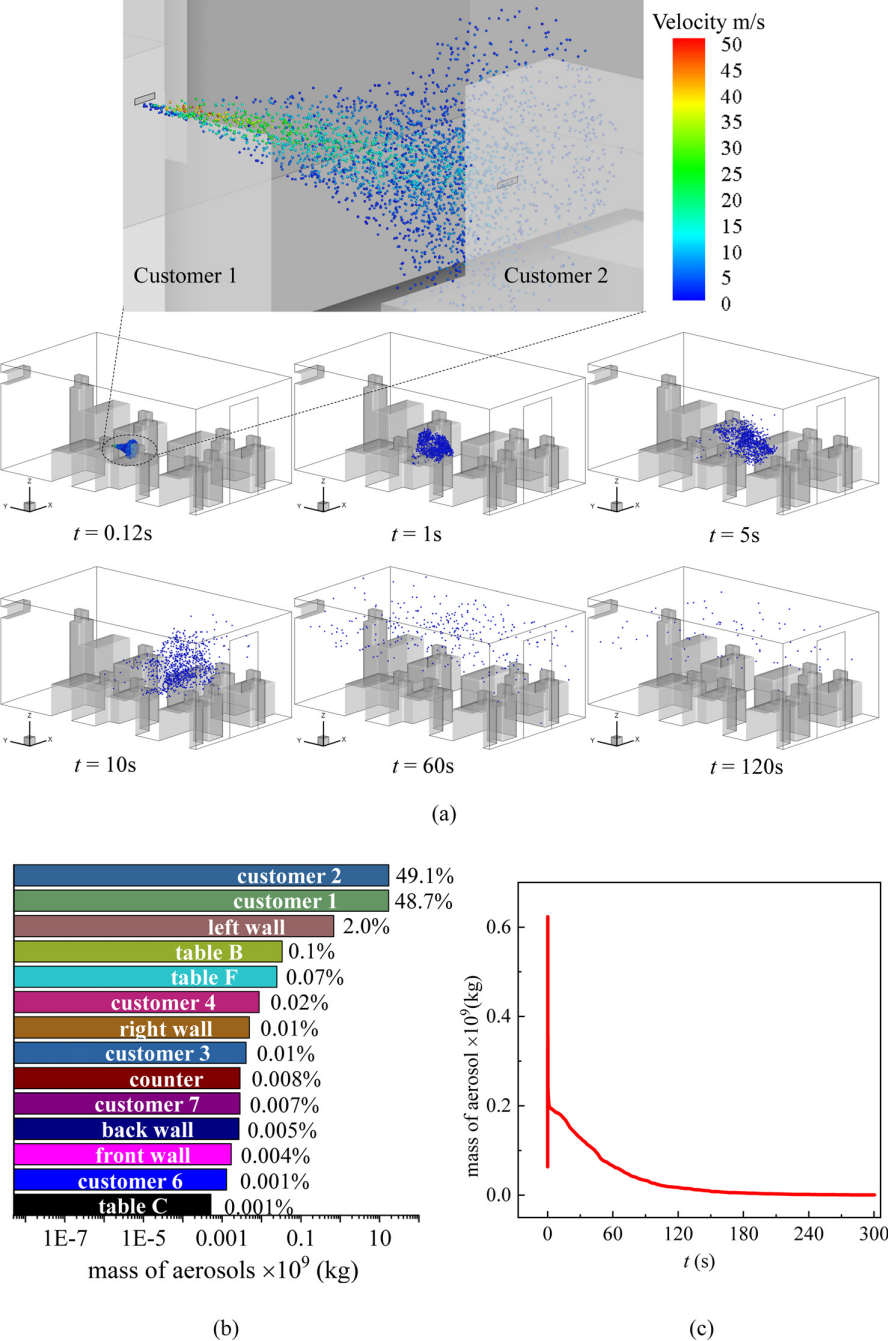
(a) Distribution of the droplets in the cafeteria when customers are dining face to face and customer 1 sneezes, (b) total mass fraction of droplets detected on the surfaces inside the cafeteria after 300 s of the sneezing, and (c) mass variation of droplets in the air with time.

**FIG. 4.**
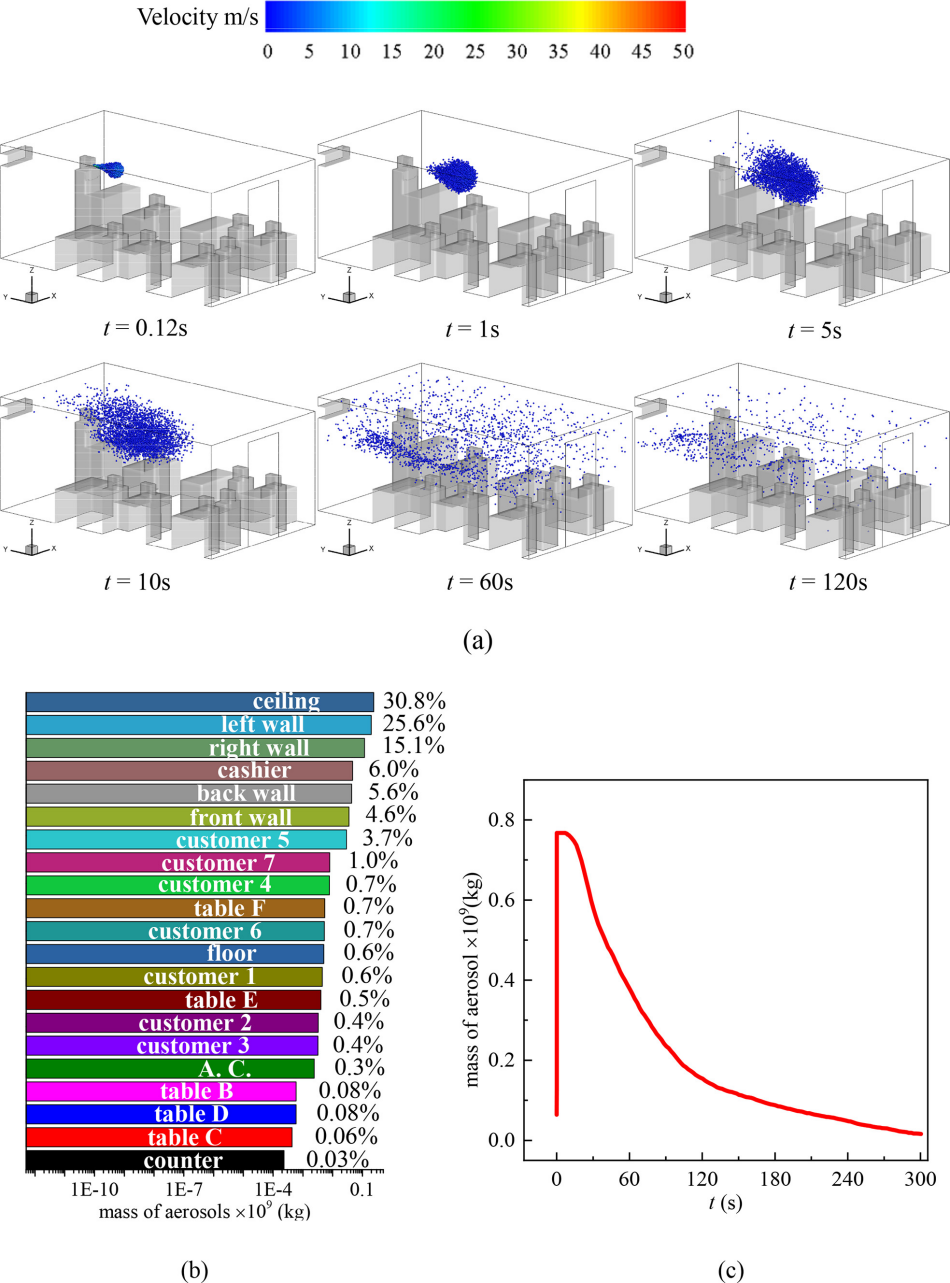
(a) Distribution of the droplets in the cafeteria when customers are dining face to face and the cashier sneezes, (b) total mass fraction of droplets detected on the surfaces inside the cafeteria after 300 s of the sneezing, and (c) mass variation of droplets in the air with time.

Two scenarios are considered for droplets transmitting inside the cafeteria when the customers are seated randomly. 7 customers and 1 cashier are inside the cafeteria. The first scenario shown in Fig. 3(a) is that a seated customer [marked as C.1 in Fig. 1(a)] sneezes at *t* = 0 s faced to another customer [marked as C.2 in Fig. 1(a)] sitting across the table [marked as Table B in Fig. 1(a)]. The second scenario [Fig. 4(a)] is that the cashier standing behind the counter sneezes at *t* = 0 s in front of a dining customer [marked as C. 5 in Fig. 1(a)] faced to the cashier.

In the scenario where customer 1 sneezes, a large number of droplets sputter onto customer 2 directly as shown in the inset of Fig. 3(a) in the first 60 s after the sneeze. At this moment, the average velocity in the *z* = 1.1 plane is 0.090 m/s and the average turbulent kinetic energy is 0.0036 m^2^/s^2^. After the cloud of sneezing droplets spreads, the movement of them is dominated by the indoor air flow. The droplets are brought by the air flow to the whole indoor environment [*t* = 60 s in Fig. 3(a)] and fall on any surfaces that are on their trajectories. Hence, the mass of droplets in the air decreases with time [Fig. 3(c)]. In this scenario, approximately 98% of the droplets are detected on the head and body of customer 1 and customer 2 [Fig. 3(b)] in 300 s after the sneeze. Sharp reduction is observed in the droplet concentration in the air as plotted in Fig. 3(c), which is contributed to the receiving of most droplets by customer 2. It is worth noting that, although customer 1 is seated in front of Table B facing the front wall, droplets are detected on the back wall of the cafeteria [Fig. 4(b)] at *t* = 34 s. Moreover, a vast number of surfaces are contaminated by the sneezing droplets of customer 1 including other tables such as Tables C and F and left, right, back, and front walls. Specifically, droplets are detected on customers 3, 4, 6, and 7 within 20 s. These customers may go somewhere else bringing the virus unconscious and cause a secondary infection. Besides, respiratory droplets can be produced during various human activities such as speaking, singing, and coughing.^10^ Deposition of droplets on surfaces indicates that both walls and tables in the cafeteria require frequent sanitizing.

Figure 4 illustrates the other scenario of virus transmission among randomly seated customers when the standing cashier sneezes. It is obvious that the location of the droplet source affects the transmission process dramatically. In this scenario, the droplets are emitted from a higher location (*z* = 1.6 m) compared to the case shown in Fig. 3. The cloud of the droplets is in the large scale of vortexes caused by the A.C. As a consequence, the circulating indoor air helps to spread the droplets quickly to the entire indoor environment [Fig. 4(b), *t* = 1–60 s]. The average velocity in the *z* = 1.6 plane is 0.15 m/s, and the average turbulent kinetic energy is 0.011 m^2^/s^2^ at *t* = 60 s. As a result, approximately 50% of the sneezing droplets float in the air in 60 s after the cashier sneezes [Fig. 4(a)]. This indicates that a higher location of sneezing droplet sources is more hazardous for indoor dining than a source at a lower location. One can walk into the cafeteria without knowing someone just sneezed 1 minute ago and be infected by the floating droplets in the air. In addition, more surfaces are contaminated by the droplets, and the decrement of the droplet concentration in air is slower compared to the scenario that sitting customer 1 sneezes. Specifically, the ceiling is the most contaminated surface due to the vortexes that carry droplets upward. In total, the ceiling and left and right walls accumulate over 70% of the droplets at *t* = 300 s. In this scenario, the surfaces of all 7 customers are contaminated by the sneezing droplets of the cashier and are likely to be infected or become a source of infection.

### C. Effect of seating

After the outbreak of COVID-19, several precautions are made to prevent the spreading of viruses in cafeterias and restaurants. Except for recommending taking out instead of indoor dining, several strategies are proposed to reduce the chance of contacting infectious sources such as keeping the social distance. One strategy in the canteens and cafeterias in our university is that the customers are recommended to take seats all facing the same direction and one table is recommended to be occupied by only one customer exclusively. Hence, the scenarios with fewer customers dining in the cafeteria are studied that no customer is dining face to face. In the two scenarios discussed in Sec. III, customers [labeled as C.1, C.2, and C.3 in Fig. 1(b)] dining in the cafeteria with 1 cashier standing is simulated. Two of the customers are facing the front wall (C.1 and C.3) and one is facing the back wall (C.2). Two scenarios that customer 1 sneezes and the cashier sneezes are considered.

**FIG. 5.**
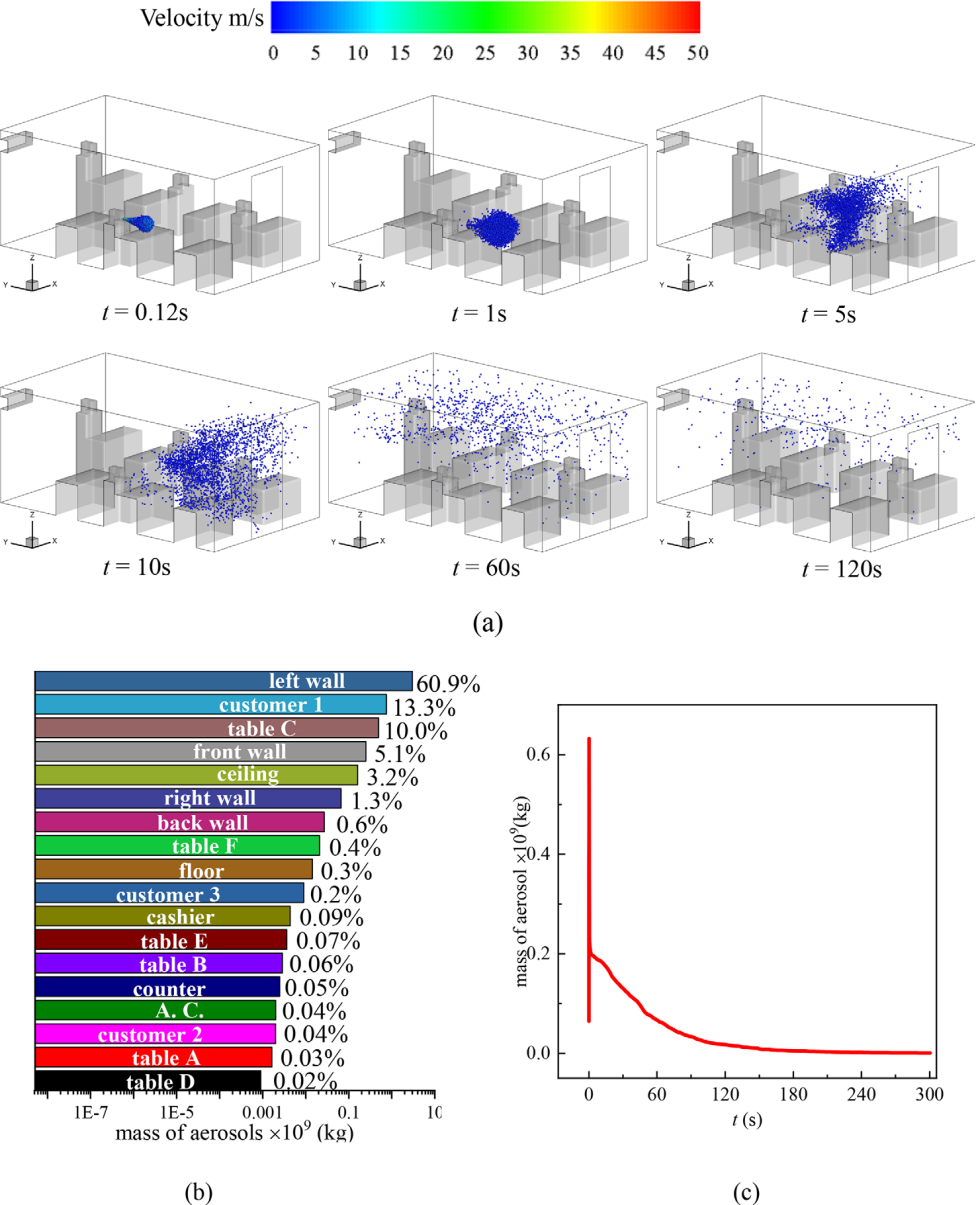
(a) Distribution of the droplets in the cafeteria when customers are not dining face to face and customer 1 sneezes, (b) total mass fraction of droplets detected on the surfaces inside the cafeteria after 300 s of the sneezing, and (c) mass variation of droplets in the air with time.

Figure 5 illustrates the transmission of droplets when customer 1 sneezes. In this situation, there is no one sitting in front of customer 1, and the droplets spread with the initial momentum from the jetting air toward the front wall. However, as there is a large scale of vortex near the front wall, the droplets are brought backward to cusotmer1 by the air. Hence, after 300 s of the sneeze, 13.3% of the droplets are detected on customer 1 [Fig. 5(b)]. Because there is no one sitting across the table facing customer 1, no other customer receives a large number of droplets as in the scenario in Fig. 3. It is worth noting that 10% of the droplets are detected on Table C, which is not the table that customer 1 seats but the table in the trajectories of the droplets. This result suggests that the unoccupied table can still be contaminated even though the customers follow the suggestion of seating separately. Sanitizing should cover all surfaces. In this context, setting screens in front of each person is beneficial for maintaining the hygienic indoor environment, which is reported in several canteens in Thailand.^46^ Various other surfaces in the cafeteria are also contaminated such as the front wall, ceiling, and floor. A small number of droplets are detected on customers 2 (0.04%) and 3 (0.2%) and the cashier (0.09%) despite the distances between them and customer 1 being much farther than the recommended social distance (6 feet).

**FIG. 6.**
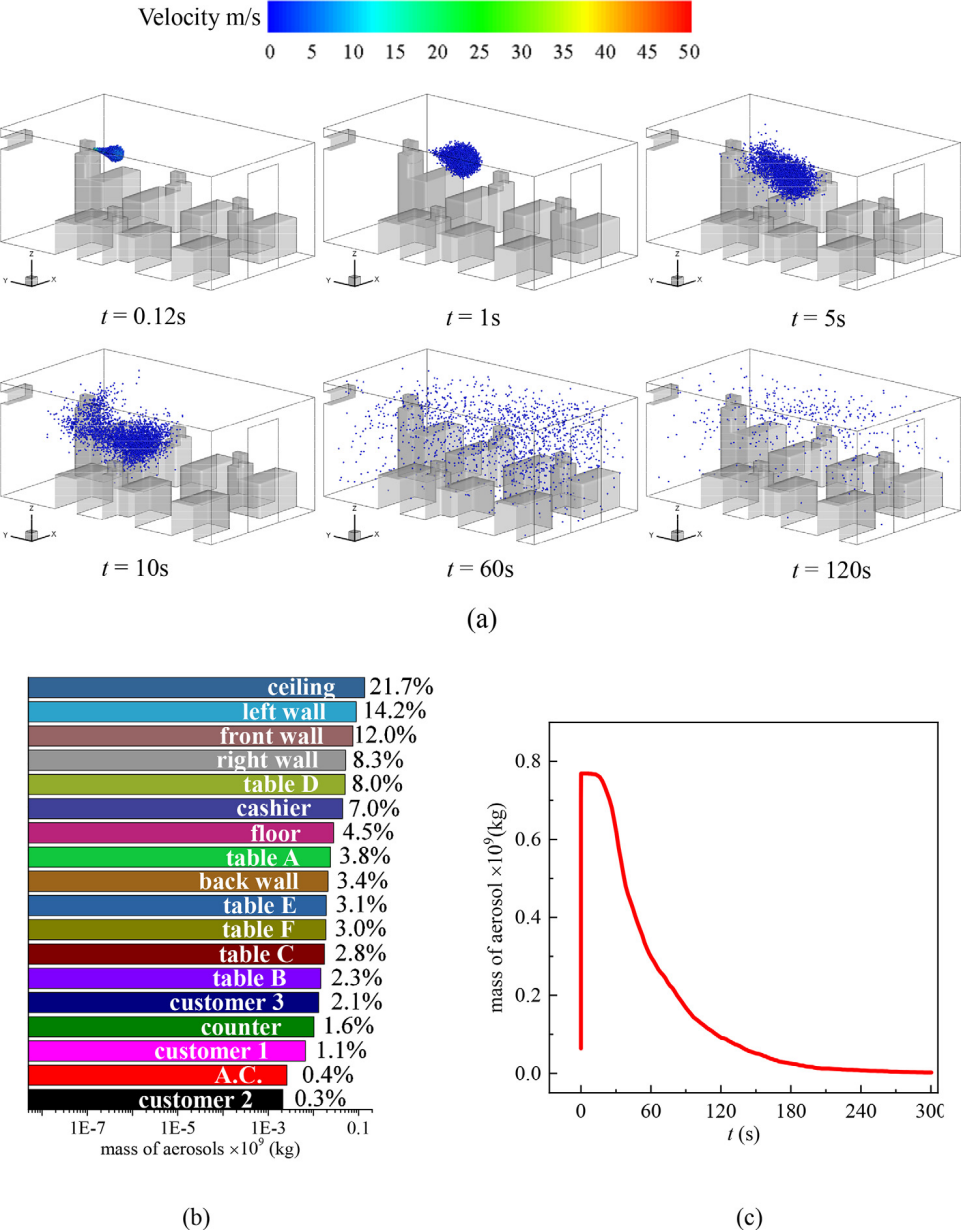
(a) Distribution of the droplets in the cafeteria when customers are not dining face to face and the cashier sneezes, (b) total mass fraction of droplets detected on the surfaces inside the cafeteria after 300 s of the sneezing, and (c) mass variation of droplets in the air with time.

Figure 6 summarizes the scenario in which the cashier sneezes and the customers are seated separately. In this case, most of the droplets are detected on the ceiling [Fig. 6(b)], and totally 56.2% of the droplets are deposited on the ceiling and left, front, and back walls in 300 s after the sneeze. The decrement of the droplet concentration in the air follows the same tendency as shown in Fig. 4(c). This indicates that the transmission of droplets in this scenario is similar to the case in Fig. 4 regardless of the seating choice of the customers. The number and location of the customers affect the local small scale of the vortexes and result in slightly different droplet deposition fractions on surfaces from Fig. 4. However, in this scenario, 8% of the droplets are deposited on Table D where customer 2 is seated. This increases the risk of contaminating the food of customer 2. Besides, in both scenarios discussed in this section, droplets are detected on all tables. This indicates that seating at separate tables and facing the same direction is not a very successful strategy in preventing the transmission of SARS-Cov-2 in an indoor environment with an A.C.

### D. Effect of the air conditioner

As discussed above, the circulation of the indoor air acts as an accomplice for the transmission of the droplets in an indoor environment. Hence, the two scenarios that the customers are seated randomly and dining face to face [Fig. 1(a)] are reconsidered under the condition without an A.C. Though the A.C. is turned off, it is impossible to stop the air flow. As long as there are temperature gradients, natural convection caused by the thermal pressure always exits.

**FIG. 7.**
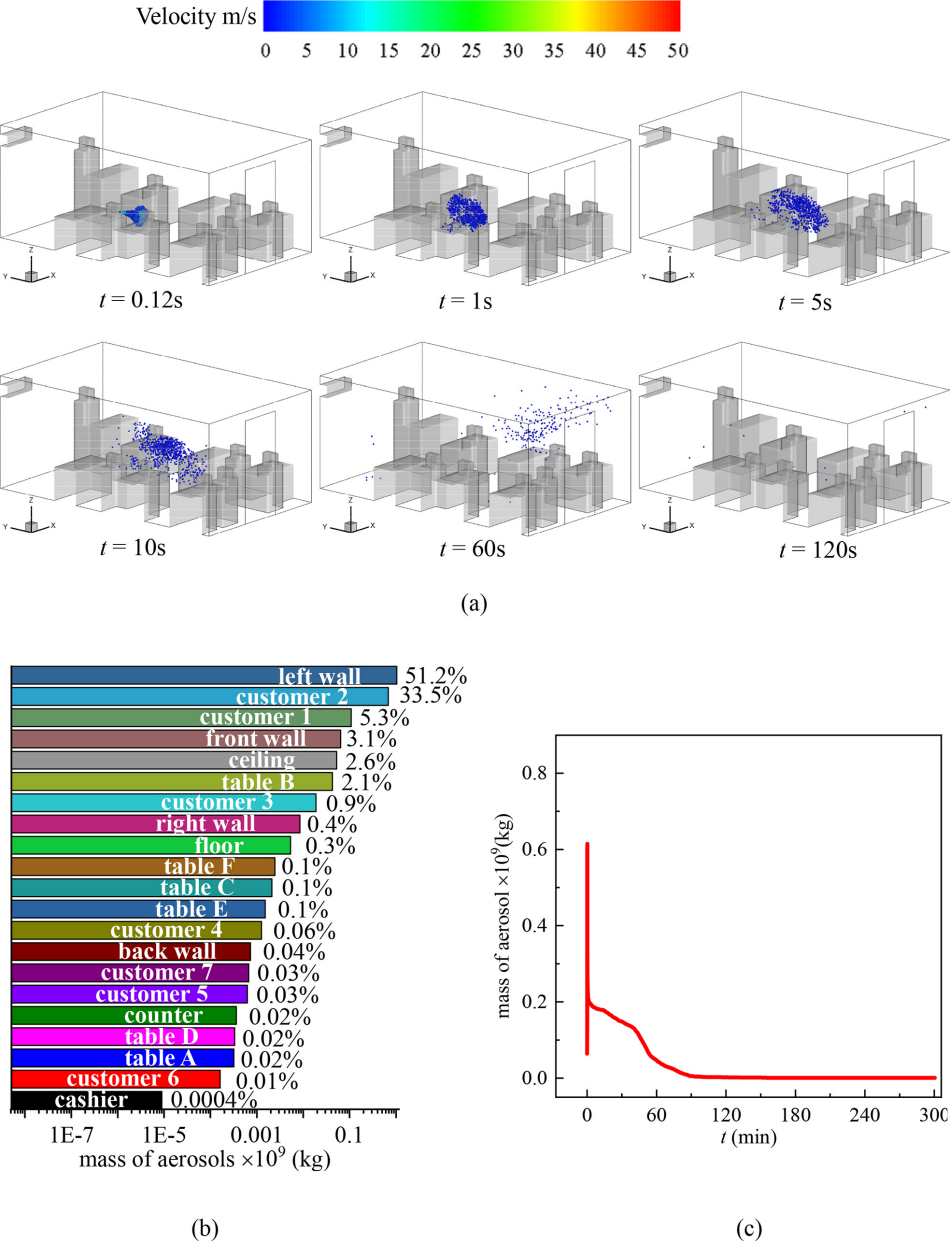
(a) Distribution of the droplets in the cafeteria without an A.C. when customer 1 sneezes and customers are dining face to face, (b) total mass fraction of droplets detected on the surfaces inside the cafeteria after 300 s of the sneezing, and (c) mass variation of droplets in the air with time.

In the scenario where customer 1 sneezes (Fig. 7) when the A.C. is turned off, most of the droplets are detected on the left wall, after 5 min of sneezing (customers 2 and 1). The droplets on these three surfaces sum to 90% of the total mass of the droplets. This indicates that the transmission of the droplets is restricted in a relatively small space in this scenario. The average velocity in the *z* = 1.1 plane is 0.0088 m/s, and the average turbulent kinetic energy is 0.00036 m^2^/s^2^ at *t* = 60 s. The droplets on customer 1 himself/herself is 5.3%, which is much less than in the scenario with a horizontal air supplying A.C. This confirms that the circulating air by the A.C. causes the deposition of droplets on the sneezing person near the wall. As shown in Fig. 2(b), the vortex caused by the A.C. results in air flow toward the back of customer 2. When the A.C is turned off, the accumulative droplets on customer 2 are also reduced. The decay in the mass of droplets in air is the fastest in all scenarios considered in this work that almost no droplets flow in the air after 120 s of sneezing. It is worth noting that 2.6% of the droplets are detected on the ceiling, and a tiny number of droplets are detected on the cashier (0.0004%). Also, 21 surfaces are detected with droplets after 300 s. This suggests that the air flow caused by natural convection is still capable of spreading the droplets all around the indoor environment.

**FIG. 8.**
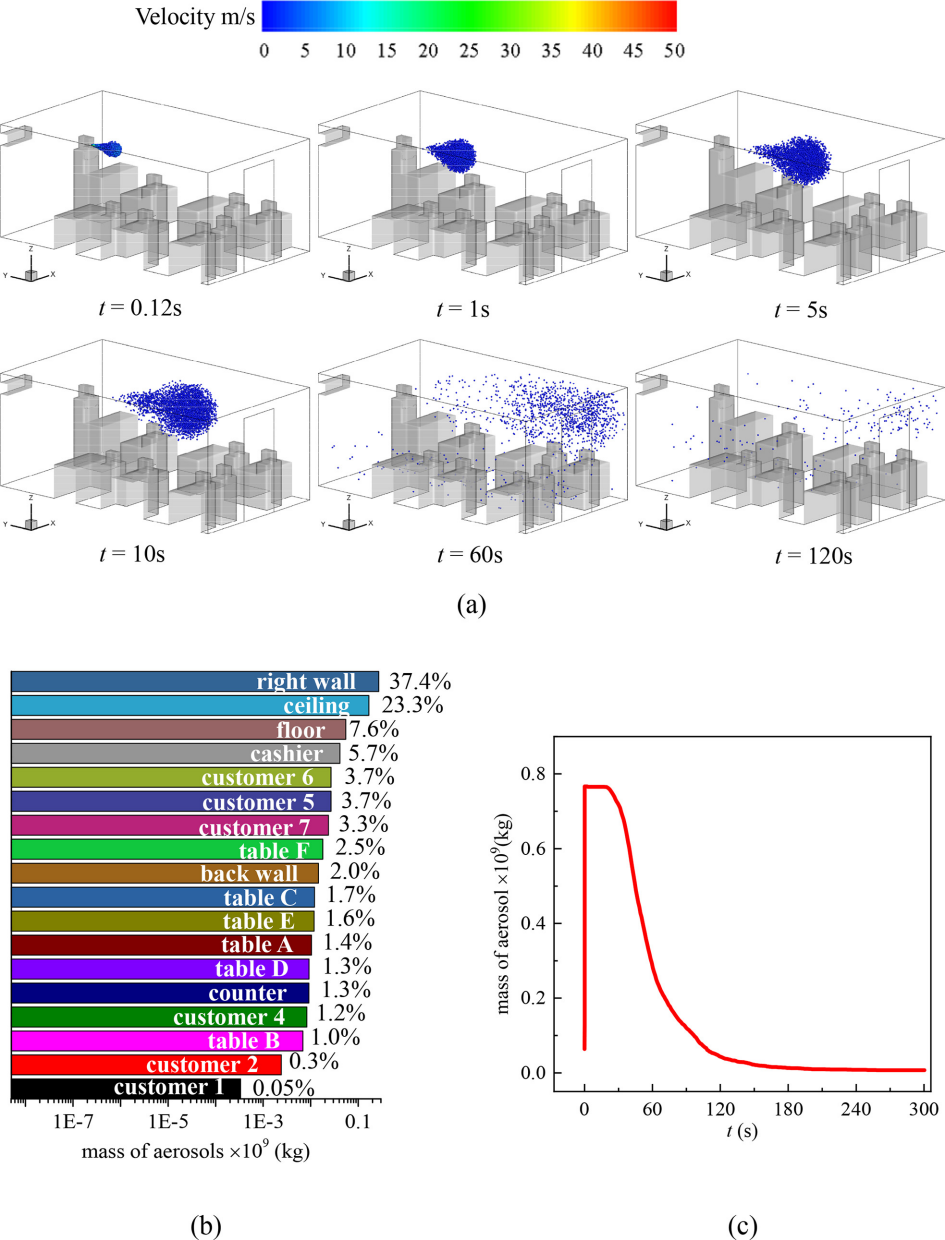
(a) Distribution of the droplets in the cafeteria without an A.C. when the cashier sneezes and customers are dining face to face, (b) total mass fraction of droplets detected on the surfaces inside the cafeteria after 300 s of the sneezing, and (c) mass variation of droplets in the air with time.

The scenario that the cashier sneezes without an A.C. is simulated, and the results are summarized in Fig. 8. Compared with Fig. 4, most of the droplets eventually deposit on the right wall, which agrees with the undisturbed trajectories of the droplets. 23.3% of the droplets are detected on the ceiling since the droplets form a cone when they are emitted out of the mouth, and some droplets are initialed with *z*-positive velocity. The decay of the droplets in the air is faster than in the scenario when the A.C. is on, and only ∼1% of the droplets still flow in the air at *t* = 240 s. The average velocity in the *z* = 1.6 plane is 0.020 m/s, and the average turbulent kinetic energy is 0.002 m^2^/s^2^ at *t* = 60 s, indicating the weaker transverse transmission of droplets. Most contaminated surfaces are on the right side of the cafeteria. There is no droplet detected on customer 3 and the left wall. However, all tables are still contaminated.

**FIG. 9.**
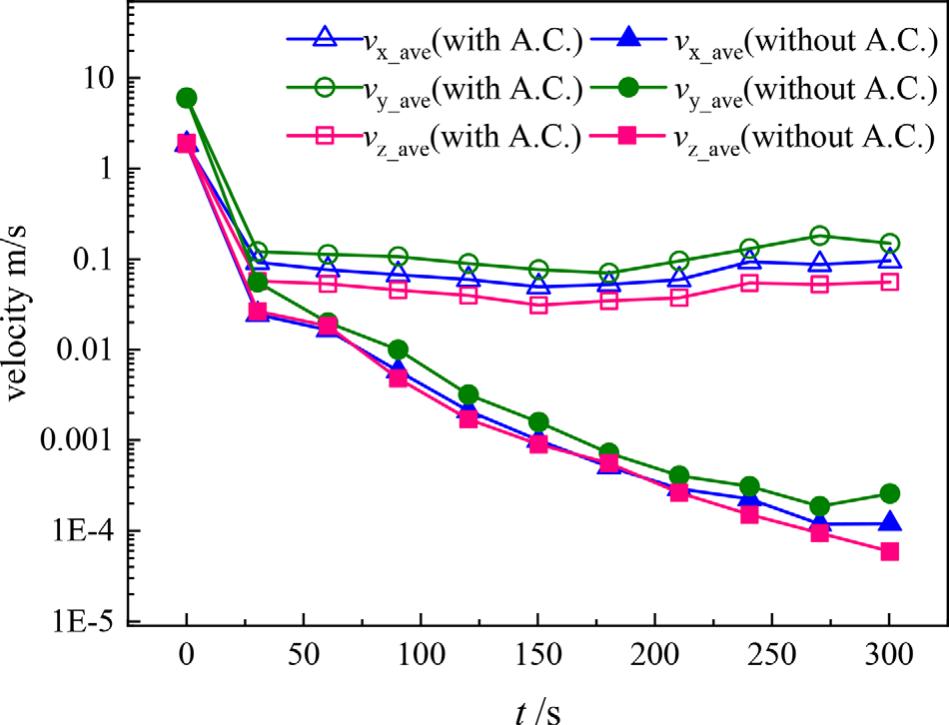
Variation of the average velocity magnitude of particles in scenarios with and without an A.C. (triangles are the *x* velocity components, circles are the *y* velocity components, and squares are the *z* velocity components).

To examine the effect of the A.C. quantitatively, the average velocity magnitude of the particles is compared in Fig. 9 under the condition that the cashier sneezes. It can be seen that the velocities of the droplets attenuate with time when there is no A.C. Averagely, the droplets lose over 99.9% of their momentum in 120 s. On the other hand, particle velocities along all *x*, *y,* and *z* directions are increased when there is a working A.C. Specifically, the increasing velocities along *x* and *y* directions make the droplets capable of traveling a longer distance horizontally inside the cafeteria. After the initial loss of droplet momentum, no obvious decrease or increase in the droplet velocities can be seen during 30 s–300 s. This confirms that the movement of the droplets is dominated by the air flow caused by the A.C. after the cloud of sneezing droplets spreads. However, it is necessary to induce clean air into an indoor space to keep the environment clean and dilute the virus concentration in air. The results in our work suggest that a horizontal supplying A.C. is an inadvisable air supply strategy in rooms where people do not wear masks. Proper air distribution should be designed for indoor activities to restrain the spread of respiratory droplets such as the vertical supplying proposed by Wang *et al.*^25^

Note that the heat and mass transfer between the sneezing droplets and the ambient air is neglected. Since the scenarios considered in our work are in winter conditions with low humidity in most cities, the amount of droplets deposited on surfaces can be lower if the evaporation of droplets is considered.^47–49^ Some droplets may become droplet nuclei that can stay airborne for a very long time. Hence, the attenuation of the droplets flowing in air may require longer time than our simulation results. However, further quantitative studies are required for determining the mass of droplets deposited on surfaces and floating in air in cities with different climate conditions.

## V. CONCLUSIONS

In this work, the transmission of respiratory droplets caused by sneezing is studied numerically in a small cafeteria with 6 tables and 1 counter. First, 2 scenarios are studied when the customers are seated randomly including face to face seating. One scenario is that a sitting customer sneezes and releases the droplets at *z* = 1.1 m, and the other scenario is that the standing cashier sneezes and releases the droplets at *z* = 1.6 m. The results show that face to face dining increases the risk of being infected by the sneeze of the person sitting across the table. ∼50% of the droplets deposit on the other customer who sits across the table in 300 s after the sneeze. When the droplets are sneezed out by the cashier at a higher location, the horizontal-supplying A.C. helps the spreading of the droplets to the entire environment and lengthens the survival time of the droplets in the air. More surfaces can be contaminated by the droplets sourcing from a higher location.

Then, a strategy that suggests that the customers sit separately, which is widely adopted by a lot of canteens and cafeterias, is examined. Both scenarios where the customer and cashier cough are discussed. Since there is no one sitting face to face during dining, the risk of being infected directly from other's sneezing droplets reduces when the sitting customer sneezes. However, the transmission of droplets when the cashier sneezes shows little difference with the scenario when customers are seated randomly and dining face to face. In this circumstance, a better way of preventing droplets is required, for example, using screens between customers.^20^

The horizontal supplying air conditioner causes vortexes inside the cafeteria and spreads the droplets transversely. So, the two scenarios with randomly seated customers are studied under the condition without an A.C. The decay of droplets in the air is faster when the A.C. is turned off in both scenarios where the customer sneezes and the cashier sneezes. The velocities of the droplets are increased by the A.C., and hence, the droplets can travel a longer distance. However, natural convection is still capable of bringing the droplets around the indoor environment. It is essential to design proper fresh air distribution strategies for indoor activities while restraining the spread of the droplets.

All scenarios studied suggest that droplets can be transmitted by air to places far away from their origin in an indoor environment. Hence, sanitizing in a cafeteria should cover all surfaces instead of sanitizing the only tables used with customers.^11^

## Data Availability

The data that support the findings of this study are available from the corresponding author upon reasonable request.
